# Porous cage-derived nanomaterial inks for direct and internal three-dimensional printing

**DOI:** 10.1038/s41467-020-18495-5

**Published:** 2020-09-17

**Authors:** Tangi Aubert, Jen-Yu Huang, Kai Ma, Tobias Hanrath, Ulrich Wiesner

**Affiliations:** 1 Department of Materials Science and Engineering, Cornell University, Ithaca, NY 14853 USA; 2 Department of Chemistry, Ghent University, Ghent, 9000 Belgium; 3 Robert F. Smith School of Chemical and Biomolecular Engineering, Cornell University, Ithaca, NY 14853 USA

**Keywords:** Organic molecules in materials science, Polymers, Synthesis and processing

## Abstract

The convergence of 3D printing techniques and nanomaterials is generating a compelling opportunity space to create advanced materials with multiscale structural control and hierarchical functionalities. While most nanoparticles consist of a dense material, less attention has been payed to 3D printing of nanoparticles with intrinsic porosity. Here, we combine ultrasmall (about 10 nm) silica nanocages with digital light processing technique for the direct 3D printing of hierarchically porous parts with arbitrary shapes, as well as tunable internal structures and high surface area. Thanks to the versatile and orthogonal cage surface modifications, we show how this approach can be applied for the implementation and positioning of functionalities throughout 3D printed objects. Furthermore, taking advantage of the internal porosity of the printed parts, an internal printing approach is proposed for the localized deposition of a guest material within a host matrix, enabling complex 3D material designs.

## Abstract (web summary)

3D printing of nanomaterials generates opportunities to create advanced materials but printing of nanoparticles with intrinsic porosity has gained only little attention. Here, the authors demonstrate printing of silica nanocages using digital light processing to fabricate hierarchically porous parts with tunable internal structure and complex shapes.

## Introduction

Three-dimensional (3D) printing techniques, or additive manufacturing technologies, have emerged as an enabling platform for the bottom-up fabrication of advanced functional superstructures covering a wide range of materials and applications, from metals and ceramics to biological tissues and organs^1–4^. Among these techniques, digital light processing (DLP) has become a versatile choice, allowing for the printing of polymeric or hybrid materials and employing simple commercial video projectors^5–9^. This technique makes use of digital micromirror devices (DMDs) to generate pre-programmed ultraviolet (UV)/blue light shapes in a plane. When combined with photosensitive resins, this allows for the rapid layer-by-layer building of macroscopic objects with arbitrary shapes and resolutions down to the micrometer scale^9–11^. Recent advances in materials science provide an extensive library of nano-sized building blocks, enabling printed materials and devices with programmable optical, magnetic, plasmonic, and catalytic properties. For light-based 3D printing purposes, nanoparticles and nanomaterials are typically used as fillers, blended with polymeric binders^6,12,13^. These composites are, however, often limited to relatively low weight fractions of the active filler. This in turn may limit the intrinsic added value of the nano-sized components. To fully exploit the potential of the combination of nanomaterials and 3D printing techniques, we developed a class of functional inks based on a photoresponsive ligand on inorganic core (PLIC) design^14^. This approach leverages prior nanomaterials research with its development of a large variety of nano-sized building blocks offering a wide range of properties. Advances in our understanding of the surface chemistry of nanostructured materials have enabled their functionalization with tailored surface ligands^15^. The confluence of nanomaterials synthesis and advanced additive manufacturing methods now allows materials scientists and engineers to combine nanoscale properties of matter with the micro- and macroscale structural control offered by 3D printing techniques.

## Results

### Formulation of the cage-based PLIC ink

**Fig. 1:**
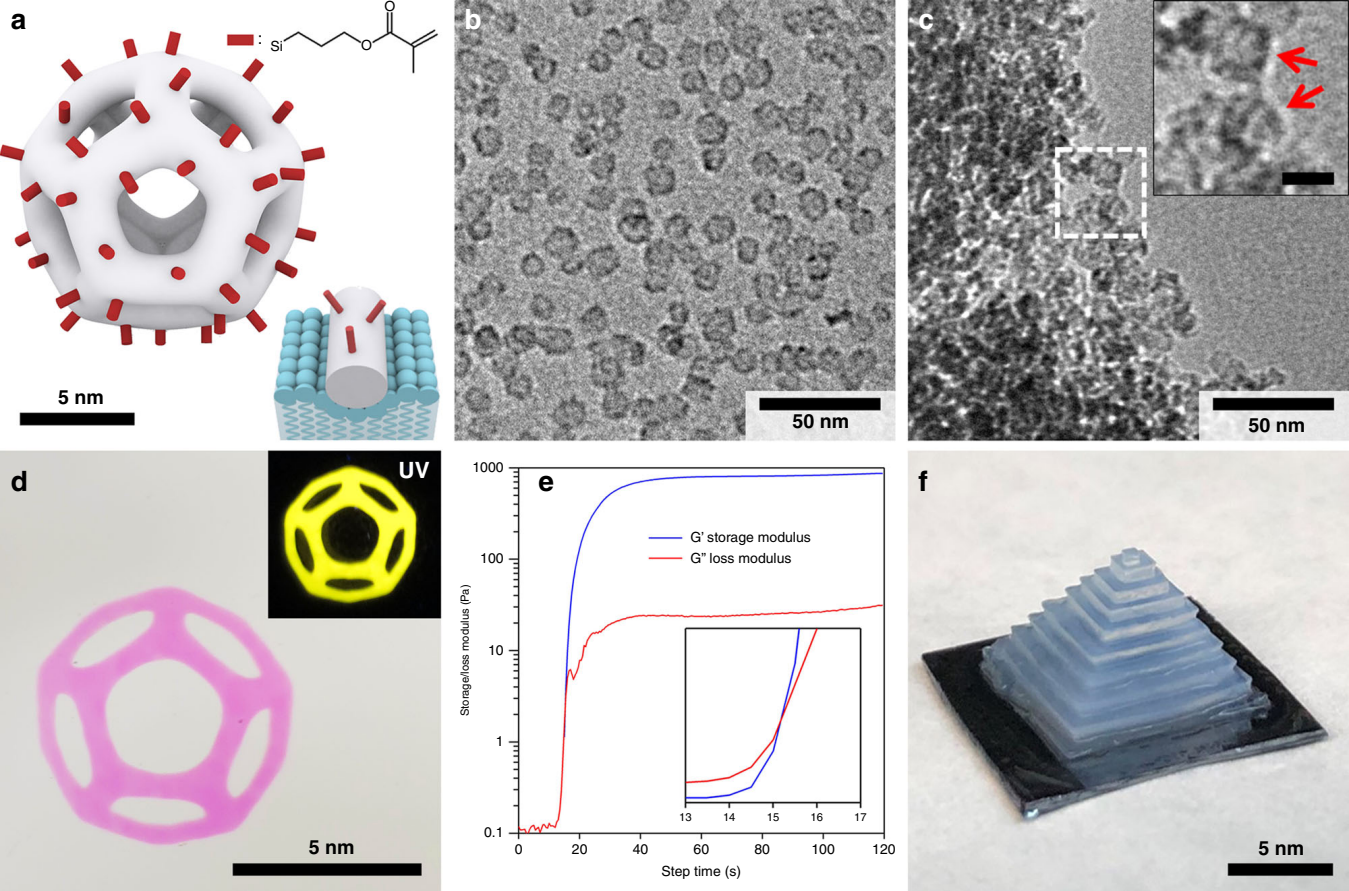
Direct printing of mesoporous parts. Illustration (**a**) and TEM image (**b**) of silica cages functionalized with methyl methacrylate groups (inset in **a**: illustration of a cage strut partially wrapped by the surfactant micelle surface, in blue). **c** TEM image of a piece of a printed part (inset: zoom in, red arrows point to clearly visible cage structures, scale bar 10 nm). **d** Photograph of a pattern printed on a glass substrate with dye-functionalized cages (inset: photograph under UV illumination). **e** Rheology measurements, including storage modulus (G’, blue line) and loss modulus (G”, red line), of the PLIC ink under UV irradiation (inset: zoom in to the region around the gel point). **f** Photograph of a multilayer 3D structure printed from the PLIC ink.

Here we make use of ultrasmall (about 10 nm) silica cages formed around cetyltrimethylammonium bromide (CTAB) micelles swollen with mesitylene and exhibiting well-defined pentagonal dodecahedral symmetry with a single, roughly 7 nm diameter internal spherical pore and 4 nm-wide window openings on each face (Fig. 1a)^16^. Their sol-gel synthesis based on hydrolysis and condensation of tetramethyl orthosilicate (TMOS) as the silane precursor provides a versatile platform for their subsequent surface functionalization with a large variety of commercially available organosilanes^17^. These cages constitute the elementary units forming a number of two-dimensional and 3D mesoporous silica materials^18,19^. Despite substantial fundamental and technological interest, few examples exist of 3D-printed mesoporous materials. To the best of our knowledge, there are no examples resulting from the use of inks derived from individual porous cage structures formulated as inks. Most examples of 3D-printed mesoporous materials are limited to extrusion techniques, often with the need for a calcination step to remove the template and generate porosity^20,21^. Ultrasmall photoresponsive ligand-stabilized porous cages provide an opportunity to formulate innovative PLIC inks for the direct assembly of predesigned macroscopic functional porous objects by means of DLP 3D printing. This approach enables the direct printing of mesoporous parts with high pore accessibility and control over the internal structure.

We functionalized silica cages with methacrylate-bearing silane groups, to allow their use as photoresponsive building blocks in the 3D printer (Fig. 1). To that end, we added 3-(trimethoxysilyl)propyl methacrylate directly to the solution after cage synthesis, but with the structure-directing surfactant micelles still present. Previous studies suggested that as part of their formation mechanism, the cage vertices and struts deform the micelle surface, with positively charged surfactant molecules wrapping the negatively charged inner silica cage surface^22^. As illustrated in the inset of Fig. 1a, this soft-templating approach allows to distinguish between inner and outer cage surfaces^23^, forcing the methacrylate-bearing silane groups to attach predominantly on the outer surface. The cages were subsequently purified by dialysis in an acidic water/ethanol mixture, which was shown to efficiently etch away the surfactant template^24,25^. Removing the cationic surfactant micelle caused the cages to precipitate, consistent with efficient cage modification with hydrophobic methacrylate groups. This is further supported by the colloidal stability of the cages after additional washing with ethanol and transfer into non-polar solvents such as toluene. The methacrylate functionalization was also evidenced by Fourier-transform infrared spectroscopy (FTIR) analyses of the ink, showing the characteristic absorption bands of methyl methacrylate groups (Supplementary Fig. 1). We scaled up the original silica cage synthesis^16^ by a factor of 30 (from 10 mL to 300 mL) to enable the 3D printing of large parts. Transmission electron microscopy (TEM) studies (see cage image in Fig. 1b from a 300 mL batch) did not show any noticeable change in cage structure from comparisons with smaller batch size-derived materials. This was encouraging and may suggest that the relatively facile water-based synthesis may be further scaled up. To formulate the photoresponsive ink for the 3D printer, we combined the methacrylate-functionalized cages with diphenyl(2,4,6-trimethylbenzoyl) phosphine oxide (TPO) as the photoinitiator. Exposing this formulation to light using a DLP UV projector (385 nm, 10 mW cm^−2^) locally triggers the polymerization of the methacrylate groups and forms robust connections between constituent silica cage building blocks, creating a mesoporous monolith with programmable geometry. The cross-linking of methacrylate groups is supported by FTIR measurements before and after light exposure (Supplementary Fig. 1), which suggested a decrease in the alkene signal as compared to the carbonyl signal.

### Direct 3D printing of mesoporous parts

We printed macroscopic patterns on glass slides using the PLIC ink as illustrated in Fig. 1d (2 min exposure, light dose 1.2 J cm^−2^). We patterned the projection of a dodecahedron (Fig. 1d), demonstrating the large scale and shape-control capabilities offered by this technique. The printed part showed high fidelity to the projection pattern (Supplementary Fig. 2). Silica cages were labeled with an organic dye during the synthesis for ease of visualization, which also made the pattern fluorescent under UV illumination (inset Fig. 1d). TEM analyses of pieces of the printed part (Fig. 1c and Supplementary Fig. 3) evidenced the porous character of the macroscopic structure. A closer look at the edge of such a piece further confirmed that the cage structure was preserved during the printing process (inset Fig. 1c). This method therefore enables the rapid deposition or 3D printing of porous materials with user-defined shape. In Fig. 1d, the parts were printed from a single projection layer, typically resulting in a thickness of about 1 mm (Supplementary Fig. 4). Photo-rheology measurements (Fig. 1e) were performed under similar conditions (1 mm ink layer, 365 nm light source, 10 mW cm^−2^ light power) as for the pattern in Fig. 1d. Although the light sources have slightly different wavelengths (385 nm vs. 365 nm), the absorption spectrum of TPO (Supplementary Fig. 5) shows that absorbance values at those wavelengths are close enough to make the experiments comparable. In Fig. 1e, the photo-rheology measurement started with a 30 s stabilization period not displayed in the plot (i.e., UV irradiation starts at *t* = 0 s). The PLIC ink showed a behavior similar to typical photo-polymerization of acrylate polymers. The gel point, defined as the crossover between the storage modulus (G′) and loss modulus (G″), was reached after about 15 s (light dose 0.15 J cm^−2^) of UV irradiation (inset Fig. 1e). In addition, the ink also shows a shear-thinning behavior (Supplementary Fig. 6), with viscosities below 0.1 Pa·s for shear rates above 10 s^−1^, well below the 5 Pa·s recommended viscosity for rapid recoating of ink layers^26^ and even below the viscosities of a typical commercial 3D printing resin. These rheological properties allow for facile bed homogenization during printing and enable the fabrication of multilayer 3D structures (Fig. 1f), even with self-supporting features (Supplementary Fig. 7). In Fig. 1f, the 3D structure was printed from ten layers of 0.9 mm in thickness using a home-built top-down setup (see “Methods” section for projector specifications). Although the photo-rheology experiment (Fig. 1e) indicated a gel point at 0.15 J cm^−2^, the light dose was set to 0.6 J cm^−2^ for each layer (i.e., 1 min exposure time) to ensure good structural cohesion of the 3D part.

**Fig. 2:**
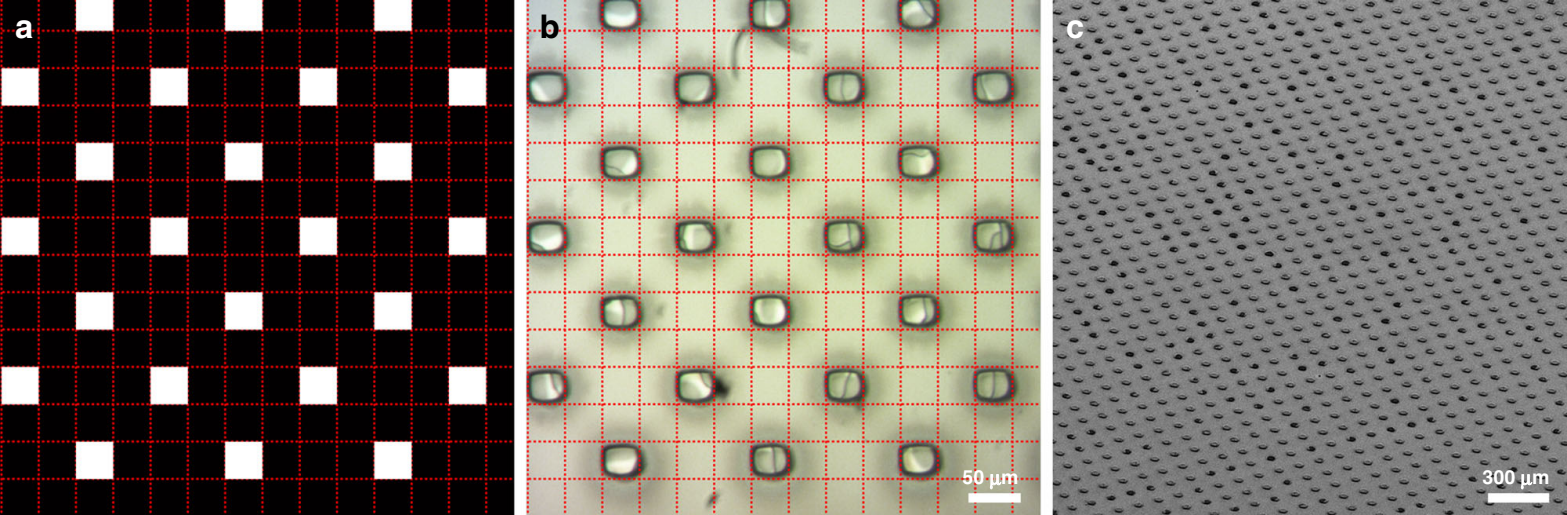
High-resolution printing. Projection pattern (**a**) and optical microscopy image (**b**) of a print at single pixel resolution (red dotted lines outline the mirrors of the DMD). **c** SEM image of a print at single pixel resolution.

With the DLP technique, a possible material-dependent limitation of the printing resolution would be light scattering from the ink itself^27^. Analysis of the as-prepared ink by UV-visible (vis) spectrometry revealed no significant scattering around the projector wavelength of 385 nm, however, with about 95% transmission through 1 cm of ink (Supplementary Fig. 8). Besides light scattering, the printing resolution for DLP is mostly hardware related. It mainly depends on the projected pixel size, which is a combination of DMD specifications and optics, and on the ability to accurately control the *z* position of the stage. The home-made setup that was used for the prints in Fig. 1d, f and Supplementary Fig. 7 is not optimized to achieve high resolution. To demonstrate that the highest resolutions can be reached using the PLIC inks reported here, we used another DLP projector, namely a Wintech PRO4710 with an orthogonal DMD for a projected pixel size of 35.5 µm (see “Methods” section for projector specifications). A thin layer of the PLIC ink was applied on a glass substrate and a pattern with single pixel resolution was projected, i.e., each of the white squares in Fig. 2a corresponds to an individual micromirror of the DMD. Observations with an optical microscope (Fig. 2b) showed high fidelity of the printed pixels with the pattern, evidencing that the hardware limit can be reached with the PLIC inks. In addition, this high resolution carries over to large areas (Fig. 2c).

### Microstructure of the cage-based printed parts

**Fig. 3:**
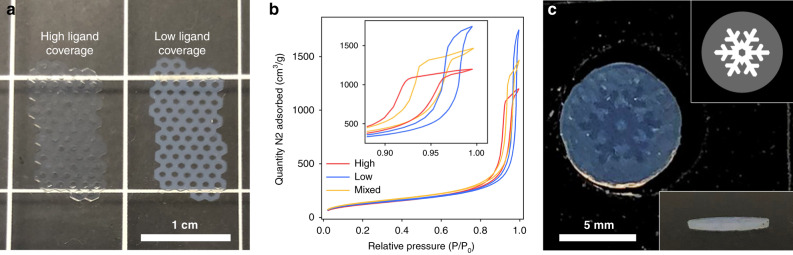
Microstructure control. **a** Photograph of parts printed with silica cages with high (left) and low (right) methyl methacrylate surface coverage. **b** Nitrogen sorption isotherms of parts printed with parent high (red line) and low (blue line) methyl methacrylate coverage materials, and with mixed (yellow line) parent materials (inset: zoom in to the hysteresis range). **c** Photograph of a part printed with a greyscale pattern (insets: greyscale pattern (top) and side view (bottom) of the part).

The combination of PLIC inks and DLP printing provided several knobs to tune the internal microstructure of the printed parts, either by changing the ligand density or by varying the light dose. Large and free-standing honeycomb structures were printed from cages with different methacrylate ligand coverage (Fig. 3a, light dose 1.2 J cm^−2^). Interestingly, adjusting the ligand coverage allowed control of the printed material density. Comparison of parts fabricated from low and high ligand-coverage PLICs revealed a positive correlation between methacrylate silane ratio and density. For example, while sharing virtually identical dimensions, as precisely measured by confocal microscopy (Supplementary Fig. 4), the parts in Fig. 3a weight 22.4 and 12.9 mg for the high-coverage and low-coverage samples, respectively. Thus, the high-coverage part is about 1.7 times denser than the low-coverage one. We attribute the difference in density to varying internal microstructures. These parts had similar specific surface areas, specifically 438 and 450 m^2^ g^−1^ for the high- and low-coverage parts, respectively, as determined by the Brunauer–Emmett–Teller method. The hystereses of the nitrogen sorption measurements (Fig. 3b) show important differences in adsorption–desorption behavior. The broader hysteresis of the high-coverage part suggests that access to mesopores is more restricted than in the case of the low-coverage part, for which mesopore access is facilitated by the presence of more interstitial space and channels.

The optical properties of the printed structure are also sensitive to the methacrylate silane ratio of the PLIC ink as illustrated by the photographs in Fig. 3a. Although after drying the high-coverage part appears relatively transparent, the low-coverage one is more translucent due to light scattering by larger pores. To induce significant Mie scattering, these larger pores likely are a few tens of nanometers in size and correspond to interparticle pores, because the intraparticle pores, i.e., cores of the cages, are <10 nm^16^. These interparticle pores are most likely formed during the drying steps, because the low ligand-coverage ink remained scatter-free during the printing process as evidenced by the absence of in-plane overgrowth (Supplementary Fig. 4). As the parts have similar specific surface area regardless of the ligand coverage, these larger interparticle pores do not contribute significantly to the total surface area. Instead, the high specific surface areas mostly derive from the intraparticle pores of the cage structure and from the microporosity of silica itself. Varying the ligand coverage of cage-based PLIC inks therefore is a powerful tool to purposefully tune the porosity and internal structure of 3D-printed objects. As a validation and reproducibility check, we prepared a PLIC ink by mixing the two cage samples in equivalent proportions. The isotherms of the resulting printed part, denoted as “mixed” (Fig. 3b), showed an intermediate behavior between those obtained from the parent high- or low-coverage materials, hence offering an additional knob to tune the microstructure of these macroscopic objects.

Alternatively to ligand density, the microstructure of the printed part can also be controlled as a function of the light dose. Indeed, using a DLP projector in video mode allows the generation of 8-bit greyscale patterns with important potential for the fabrication of materials with advanced properties^28–30^. In Fig. 3c, the single layer part was printed from a high ligand density ink using a greyscale pattern (top inset Fig. 3c). The snowflake shape has a lightness of 100% (light dose 1.2 J cm^−2^) and the surrounding disc has a lightness of 40% (light dose 0.2 J cm^−2^). Different light doses resulted in different density and optical transmission between the domains of the printed part. The domain that received a light dose of 1.2 J cm^−2^ appears more translucent like the high ligand density part in Fig. 3a, whereas the domain that received a lower light dose exhibits much more light scattering due to more interparticle porosity as described above. The side view of the part confirmed that these differences did not result from thickness variations (bottom inset Fig. 3c). The difference in light transmission between domains that received different light doses can be further evidenced from printed piece shown in Supplementary Fig. 9. Thus, using greyscale patterns allows tuning the microstructure within an individual layer. This result illustrates one of the advantages of DLP printing as compared to other techniques, such as conventional photolithography using physical masks, which may not be able to modulate light doses throughout individual layers.

If desired, the organic components of the printed parts can be removed by calcining the structures (550 °C for 6 h in air) to produce fully inorganic parts, while preserving their macrostructure and their microstructure (Supplementary Fig. 10). Calcination does lead to shrinkage, however, with the printed parts with high and low coverage resulting in a linear isotropic shrinkage of 17% and 12% (Supplementary Fig. 4), together with a weight loss of 29% and 18%, respectively. This shrinkage and weight loss result from the thermal decomposition in air of the organic fragment of the methacrylate silane as well as, to some extent, from the further condensation of silanol groups, which releases one water molecule per Si-O-Si bond formed. The fact that printed parts retain their macroscopic shape after removal of the organics suggests that the formation of such Si-O-Si bridges between cages, promoted by either the thermal treatment or just the proximity between the cages, may play a role in material cohesion. The differences in shrinkage and weight loss between the high- and low-coverage samples are consistent with the different ratios of organic to inorganic components. Nitrogen sorption measurements before and after calcination showed very similar hysteresis shapes, suggesting that microstructure and mesopore access remained unchanged (Supplementary Fig. 10). This is also supported by TEM observations of calcined parts, which showed very similar morphologies as before calcination (Supplementary Fig. 3). In addition, the specific surface area increased to 646 and 639 m^2^ g^−1^ for the high- and low-coverage materials, respectively (as compared to 438 and 450 m^2^ g^−1^ before calcination).

### Positioning of functionalities in cage-based printed parts

**Fig. 4:**
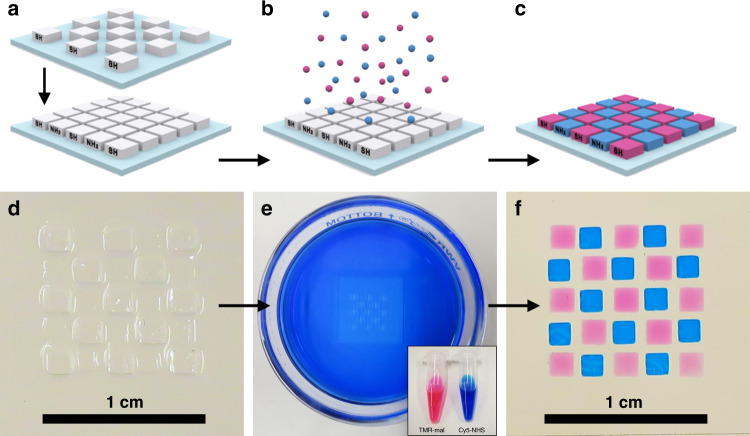
Positioning of functionalities within printed structures. Illustrations (**a**–**c**) and photographs (**d**–**f**) of the spatially controlled deposition of silica cages with different functional groups and the selective binding of organic dyes. Two independent layers of cages functionalized with either thiol or amine groups are printed successively in a shifted checkerboard pattern (**a**, **d**). The pattern is then exposed to a mixture of TMR-mal and Cy5-NHS dyes (**b**, **e**), which bind selectively to the thiol and amine functionalized tiles, respectively (**c**, **f**). Between each step, the pattern was washed with methanol.

**Fig. 5:**
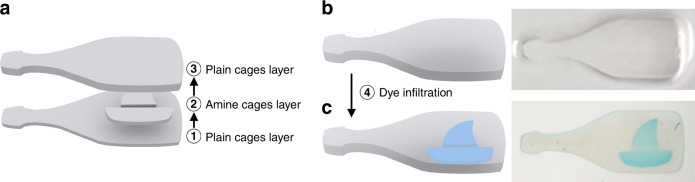
Accessibility of functionalities within printed structures. A ship pattern made of amine and methacrylate-functionalized cages was printed between two layers of plain cages only functionalized with methacrylate ligands and forming a bottle shape (**a**). In the resulting part (**b**), the “ship in a bottle” is invisible. After immersion in a Cy5-NHS solution, however, the dye penetrates the structure and bind selectively to the amine groups, revealing the ship after additional washing (**c**).

The ability to directly print 3D shapes of porous materials with controlled structure, complemented by the large choice in possible cage surface functional groups, offers a versatile platform to realize advanced materials with innovative designs. Specific functionalities can thereafter be implemented with high spatial control in complex 3D architectures. As a proof-of-concept experiment (Fig. 4), methacrylate-functionalized cages were modified post synthesis, but prior to printing, with either thiol or amine groups, using (3-mercaptopropyl)trimethoxy-silane (MTPMS) or (3-aminopropyl)trimethoxy-silane (APTMS), resulting in two different inks. We printed two successive layers on a modified glass substrate, to create a checkerboard pattern of about 1 mm in thickness, with spatially resolved thiol and amine functionalized tiles, simply by replacing the ink in the printer vat between the two layers (Fig. 4a, d). To improve adhesion of the printed parts and avoid moving of the pattern during ink replacement and intermediate washing steps, the glass substrate was functionalized with methacrylate groups prior to printing. Glass slides were first treated with a strong base to increase the surface density of silanol groups and promote subsequent condensation with methacrylate silane. This process was monitored by contact-angle measurements (Supplementary Fig. 11). An increase in the hydrophobicity of the substrate confirmed grafting with methacrylate groups. To demonstrate the spatially programmable chemical functionality of the two printed materials, we then immersed the structure for 1 h under gentle shaking (about 50 r.p.m.) in 5 mL of a mixture of 200 nmol of tetramethylrhodamine-6 C2 maleimide (TMR-mal, pink dye in Fig. 4b, e) and 200 nmol of sulfo-cyanine5 succinimidyl ester (Cy5-NHS, blue dye in Fig. 4b, e) in dimethyl sulfoxide (DMSO). Thanks to the porous nature of the printed material (i.e., without requiring a calcination step), these two dyes penetrated the structure and selectively bound to specific tiles based on their respective click chemistry (Fig. 4c, f). This result is in line with nitrogen sorption results, suggesting high pore accessibility. To demonstrate that larger molecules such as organic dyes can diffuse throughout the as-printed structures, we fabricated a multilayer 3D object in the shape of an amine functionalized ship printed inside a bottle (Fig. 5a). Thus, the functionalities in this part were fully surrounded by plain cages (i.e., only functionalized with methacrylate ligands). Although initially invisible (Fig. 5b), the ship appeared after immersion in a solution of Cy5-NHS dye, which was able to infiltrate the structure and bind selectively to the amine groups (Fig. 5c). This approach therefore enables the spatially pre-determined distribution of accessible functionalities throughout the whole volume of the printed part, rather than just on the surface. This is enabled by using cage structures and strongly benefits from the facts that (i) the methacrylate functionalization prior to template removal leaves the inner cage surface available for orthogonal functionalization and (ii) pore accessibility does not require any calcination step that would eliminate the functionalities. We note that this approach can be extended to other click-type reactions and to biological applications, such as bioassays, for the detection of specific peptides or proteins for instance, where selectivity can be addressed as a function of chemical functionality and controlled porosity for size-exclusion strategies. As the porous particles are ultrasmall, and their inner and outer surfaces can be orthogonally functionalized prior to printing, this provides access to high information density materials, e.g., for multiplexed detection of various analytes and substrates.

### Internal printing in cage-based printed parts

**Fig. 6:**
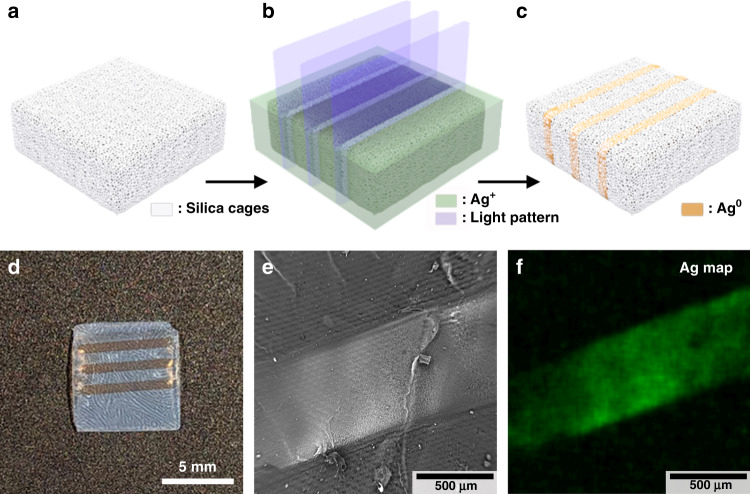
Internal printing. **a**–**c** Illustration of the internal printing of silver within a primary part printed with porous silica cages. First, a block of silica cages without additional functionalization is printed (**a**). The block is immersed in a solution of silver nitrate and photoinitiators. A light pattern in the shape of slabs is then projected onto this block (**b**), resulting in the localized reduction of Ag^+^ ions into Ag^0^ (**c**). **d** Photograph of the resulting block of silica cages exhibiting three slabs of metallic silver. Backscattered electron-based SEM image (**e**) and EDS map (**f**) of a silver slab embedded in the porous silica matrix.

Making use of the intrinsic and readily accessible porosity of the printed cage-based materials, we also propose an internal printing approach as another way of implementing functionalities within printed parts. Here, the pores of an already printed part serve as a scaffold for the subsequent printing of a second material directly within the first. To demonstrate this concept, we printed a second metal structure within the pores of a first printed silica structure. As illustrated in Fig. 6, a block of silica cages was first printed as described before and then soaked for 30 min in a solution of silver nitrate (0.1 M in 10 : 1 v/v ethanol : toluene) and two photoinitiators, namely TPO (0.05 M) and Darocur 1173 (0.5 M). In this case, TPO acts as a sensitizer for Darocur 1173, which serves as the electron donor for the reduction of Ag^+^ to Ag^0 ^^8^. A light pattern was then projected in the form of three slabs to locally reduce silver (Fig. 6a–c). The silver slabs, visible by the naked eye (Fig. 6d), were further observed by scanning electron microscopy (SEM), showing high contrast in backscattered electron-based imaging (Fig. 6e), and matching well with elemental mapping by energy-dispersive X-ray spectroscopy (EDS; Fig. 6f). The fact that silver is not quantitatively found outside of the slab pattern by EDS analysis indicates that detected silver inside the slab is indeed in the form of metallic Ag^0^ particles, which remained embedded within the pores, and unlike unreduced Ag^+^ ions, could not escape from the original silica block during washing steps. The analysis of the cross-section by EDS also confirmed the presence of silver inside the volume of the slab (Supplementary Fig. 12). The internal printing of silver structures is demonstrated here in a single thick layer. Several approaches can be conceived for its implementation in multilayer prints, either by changing the solution in the ink vat between each layer in a bottom-up projection setup or by progressively re-immersing a fully printed object in a top-down projection setup (see schematics in Supplementary Fig. 13). The cure depth (or reduction depth in the case of a metal structure) can then be controlled by using a light absorber or by tuning the light dose, hence offering free-form capability on the second material in both *xy* and *z* directions. Alternatively, one can also envision using femtosecond lasers to print the guest material with high spatial resolution inside a printed cage-based mesoporous part, similar to the direct laser writing of metal and metal halide structures in glasses or polymer gels^31,32^.

## Discussion

The 3D printing of a second material within a 3D-printed mesoporous silica block opens a broad and versatile opportunity space. Through this approach, the two materials are entangled with each other, which means that the structure of the scaffold or host material will influence the structure and therefore properties of the guest material. This entanglement also allows for interactions, such as charge transport, between the two materials. These interactions may even benefit from the high interfacial area between them. Deliberately varying the porosity of the silica host and hence the interconnectivity of the guest materials thereafter is expected to modulate charge and ion transport properties throughout the printed structure. Filling the cages with electrochemically or optically active guest materials further creates an interesting class of “confined but connected” materials with important practical implications, e.g., for the design and discovery of battery or photovoltaic materials with programmable mesostrucure and hierarchical architectures^33^. This approach can readily be extended to a large variety of materials (e.g., semiconductors and metal oxides) offering a wealth of unique properties. For instance, printing two different catalytic materials within a porous scaffold could result in a highly tunable platform with controlled symmetry and flux for tandem catalysis applications^34^. The whole 3D printing toolbox can thereafter be put to practice for the internal printing and positioning of active centers within the host scaffold with a great degree of freedom.

In conclusion, the design of PLIC inks based on functional porous nanomaterials and their combination with 3D printing techniques, such as DLP, offer an innovative playground to engineer ever more sophisticated devices with increased capabilities. By formulating inks from intrinsically porous nano-sized building blocks, we performed first proof-of-principle experiments for the direct printing of mesoporous parts, which in turn enabled an internal printing approach. The versatile functionalization of the silica cages further allowed to control the internal microstructure and functionalities of the printed structures. We foresee that this approach will enable a number of device architectures with important potential for sensing, catalytic, or energy applications^35^, which so far were not directly accessible with conventional material processing techniques.

## Methods

### Methacrylate-functionalized silica cages

Silica cage synthesis was adapted from a previously reported method^16^. The synthesis was scaled up as compared to the original method, simply by multiplying all molar quantities by a factor 30. In a 500 mL flask, 3.75 g of CTAB was dissolved in 300 mL of water at 30 °C. Three hundred microliters of ammonia (2 M in ethanol) and 3 mL of mesitylene were added, and the mixture was allowed to stir (800 r.p.m.) for 4 h. Three milliliters of TMOS was then added slowly and the reaction was left to proceed for one day at 30 °C. For initial experiments (samples denoted as high ligand-coverage materials), 3 mL of 3-(trimethoxysilyl)propyl methacrylate (methacrylate silane) was added slowly and the reaction was left to proceed for another day. For samples denoted as low ligand-coverage materials, only 1.2 mL of methacrylate silane was added. For purification of the functionalized cages, the synthesis solution was first dialyzed in 2 L of water : ethanol : acetic acid (v/v 500 : 500 : 7) under slow stirring, changing the solution four times over the course of 2 days, then dialyzed in 2 L of water, changing the solution three times over the course of 4 days. The cages were then collected by centrifugation (7000 × *g*, 10 min) and redispersed in 150 mL of ethanol. The cages were further washed using spin filters (VivaSpin, 100 kDa molecular weight cut-off) and centrifugation (2400 × *g*), until concentrated to a total volume of 4.5 mL. The cage solution was finally diluted with 4.5 mL of toluene and used as made. The twofold dilution with toluene was found to prevent gelation of the ink due to uncondensed silanol groups in sol-gel-synthesized silica and hence increased ink shelf life. Toluene was chosen to also help dissolution of the photoinitiator in the ink. We finally note that from results of independent studies using the same photoinitiator, other solvents may be used such as propylene glycol monomethyl ether acetate^14^.

### In-situ dye functionalization of cages

For the direct fluorescent labeling of cages during the synthesis (Fig. 1d), the synthesis was downscaled 15 times. A dye-silane conjugate was prepared 1 day prior to the synthesis by mixing 0.45 µmol of TMR-mal and 1.74 µL of MPTMS in 67 µL of DMSO under inert atmosphere. This dye-silane conjugate was added immediately after the TMOS addition during the cage synthesis. The remainder of the procedure was kept unchanged.

### Thiol and amine functionalization

For the post-synthesis modification of cages, either 83.2 µL of MTPMS or 78.4 µL of APTMS (for thiol and amine functionalization, respectively) was added to 4 mL of the cage solution. The mixture was allowed to stir for 3 days at room temperature and then used as is.

### Modification of glass substrates

For the preparation of methacrylate-functionalized substrates, cover glass slides (1.8 × 1.8 cm) were first activated by soaking overnight in a petri dish containing 5 mL of a 0.2 M NaOH solution in water. The substrates were then rinsed with water and soaked another night in a mixture of 3.5 ml ethanol, 0.5 mL NH_4_OH (28–30% in water), and 1 mL of methacrylate silane. The substrates were finally rinsed with ethanol and dried with nitrogen.

### Three-dimensional printing

PLIC inks for printing were formulated by dissolving the photoinitiator, TPO (7 mM, 0.3 wt%), in the silica cage solutions. The inks were bubbled with nitrogen for 2 min before usage to remove oxygen. Except for the high-resolution print (Fig. 2), all samples were printed with a home-made top-down DLP 3D printer using a Wintech PRO4500 projector based on a DLP4500 WXGA (912 × 1140) diamond pixel DMD, equipped with a 92 mm working distance lens (projected pixel size of 50 µm) and a 385 nm light-emitting diode (LED; output power 10 mW cm^−2^). For printing on glass substrates (Figs. 1d, 4, and 5), substrates were placed on top of a fitted piece of Si wafer in a 1.9 × 1.9 cm square shaped Teflon dish. The ink (280 µL) was added to the dish and the part was printed as a single layer by projecting the light pattern for 2 min. For the printing of free-standing parts (Figs. 1f, 3, and 6 and Supplementary Figs. 7, 9, and 10), the samples were printed directly on top of the Si wafer, resulting in easy detachment after drying. Printed samples were washed with methanol for 24 h and dried with supercritical carbon dioxide dryer (Leica CPD300)^14^. For the high-resolution print (Fig. 2), a thin layer of ink was applied on a glass slide using a TQC Sheen cube applicator with a gap of 38 µm and a single pixel pattern was projected using a Wintech PRO4710 projector based on a DLP4710 1080p (1920 × 1080) orthogonal pixel DMD, equipped with a 92 mm working distance lens (projected pixel size of 35.5 µm) and a 385 nm LED (output power 10 mW cm^−2^). To prevent quick drying of the ink in such very thin layers, the methacrylate-functionalized cages were dispersed in a mixture of ethanol : *N*-methyl-2-pyrrolidone (1 : 1 vol%) instead of ethanol : toluene. The cage concentration in the ink was kept the same as for other inks.

### Characterization methods

TEM images were acquired using a FEI Tecnai T12 Spirit microscope operated at an acceleration voltage of 120 kV. For the TEM analysis of the methacrylate-functionalized silica cages (Fig. 1b), the as-prepared solution was diluted by a factor of 1000 in toluene, then 10 µl of this solution was dropped onto a carbon coated TEM grid and the excess solution was blotted with a filter paper. For the TEM analysis of the printed part (Fig. 1c and Supplementary Fig. 3), small pieces were roughly crushed, then the resulting powder was lightly sprinkled on a carbon coated TEM grid and excess material was removed by gentle air blowing. SEM (backscattered electron and secondary electron) images and EDS elemental maps were acquired using a Tescan Mira3 field-emission SEM operated at an acceleration voltage of 10 kV. Nitrogen sorption isotherms were acquired using a Micromeritics ASAP 2020. Confocal microscopy images for dimensional analyses were acquired using a Keyence VK-X260 laser-scanning confocal microscope. FTIR spectra were acquired using a Perkin Elmer Spectrum 1000 spectrometer. UV-vis transmission spectra were acquired using a Perkin Elmer Lambda 365 spectrometer. Contact-angle measurements were performed using a Rame-Hart 500 Goniometer, with MilliQ water dropped on either as-received glass slides or freshly prepared modified substrates. Rheology measurements were performed with a DHR3 rheometer from TA Instruments, using parallel plates of 20 mm in diameter and a Omnicure Series 1500 light source with a 365 nm filter (output power 10 mW cm^−2^). Storage and loss moduli were measured at a shear rate of 60 s^−1^. Optical microscope images were acquired using a Leitz Laborlux microscope with a 10× air objective.

## Supplementary information

Supplementary Information

## Data Availability

The data sets generated and analyzed during this study are available from the corresponding author on reasonable request.
