# Supplementary material for: Porous cage-derived nanomaterial inks for direct and internal three-dimensional printing
Source: Nat Commun. 2020 Sep 17;11:4695. doi: 10.1038/s41467-020-18495-5 (PMC7499254; doi:10.1038/s41467-020-18495-5)
Supplement: Supplementary file 1 — Supplementary Information [file 41467_2020_18495_MOESM1_ESM.pdf]

Supplementary Information

Porous Cage-derived Nanomaterial Inks for Direct and Internal Three-Dimensional Printing

Aubert *et al.*

Authors

Tangi Aubert[1,2†], Jen-Yu Huang[3†], Kai Ma[1], Tobias Hanrath[3]*, and Ulrich Wiesner[1]*

Affiliations

[1] Department of Materials Science and Engineering, Cornell University, Ithaca, NY 14853, USA.

[2] Department of Chemistry, Ghent University, Ghent, 9000, Belgium.

[3] Robert F. Smith School of Chemical and Biomolecular Engineering, Cornell University, Ithaca, NY 14853, USA.

† These authors contributed equally: Tangi Aubert, Jen-Yu Huang

* email: ubw1@cornell.edu, tobias.hanrath@cornell.edu

Content

- FTIR analysis of the ink
- Pattern vs. printed part overlay
- Additional TEM images
- Confocal microscopy and line profiles
- Optical characterization of photoinitiator and ink
- Additional true 3D structures and greyscale printing
- Effect of calcination on macrostructures
- Additional nitrogen sorption measurements
- Contact angle measurements
- Cross section EDS analysis
- Illustration of 3D capable internal printing approaches
- Additional reference

Supplementary Figures

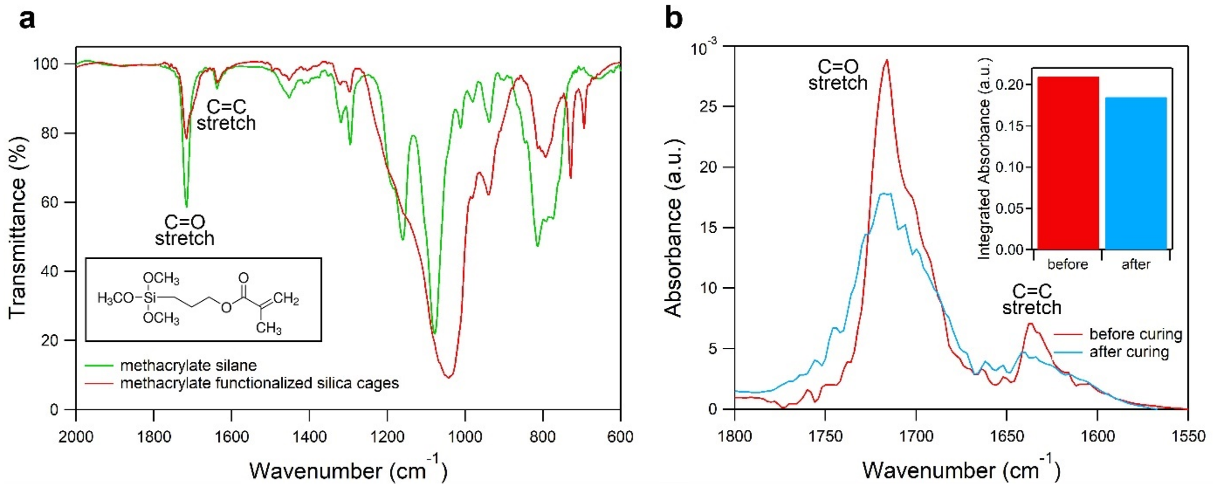

Supplementary Fig. 1. **a**, FTIR transmission spectra of the neat methacrylate silane (green line) and the as-prepared methacrylate functionalized silica cage ink (red line) without photoinitiator (inset: chemical structure of 3-(trimethoxysilyl)propyl methacrylate, the methacrylate silane), showing the characteristic absorption bands from the stretching vibration of the carbonyl, C=O, group at 1720 cm^{-1}, and the stretching vibration of the C=C double bond at 1630 cm^{-1}.[1] **b**, FTIR absorption spectra of the main C=O and C=C bands before (i.e. ink without photoinitiator, red line, same as the red curve in **a**), and after curing (i.e. printed part grounded into a powder, blue line). In **b**, the absorption spectra were normalized based on the integrated absorbance (rather than simple peak intensity) of the C=O band (from 1660 cm^{-1} to 1780 cm^{-1}) to account for the broadening of the band. The C=C absorbance band was then integrated (from 1550 cm^{-1} to 1650 cm^{-1}) and the values are reported in the inset of **b**, exhibiting a decrease as expected from polymerization.

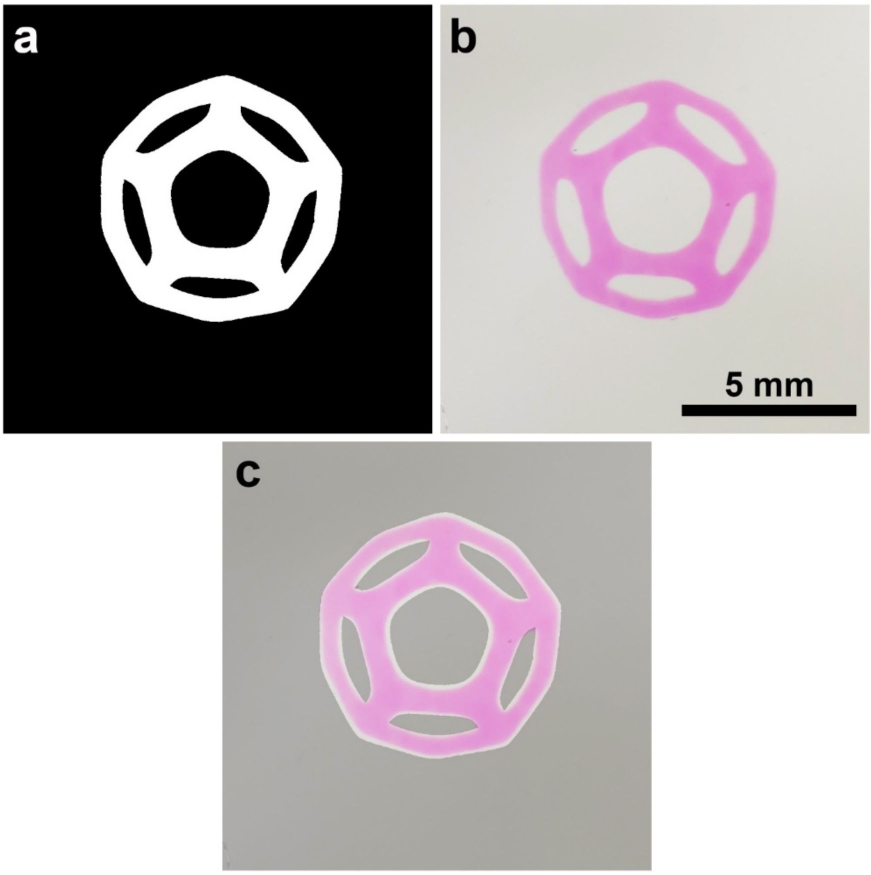

Supplementary Fig. 2. Example of a projection pattern (**a**), photograph of the resulting printed part (**b**), and overlay of the two (**c**).

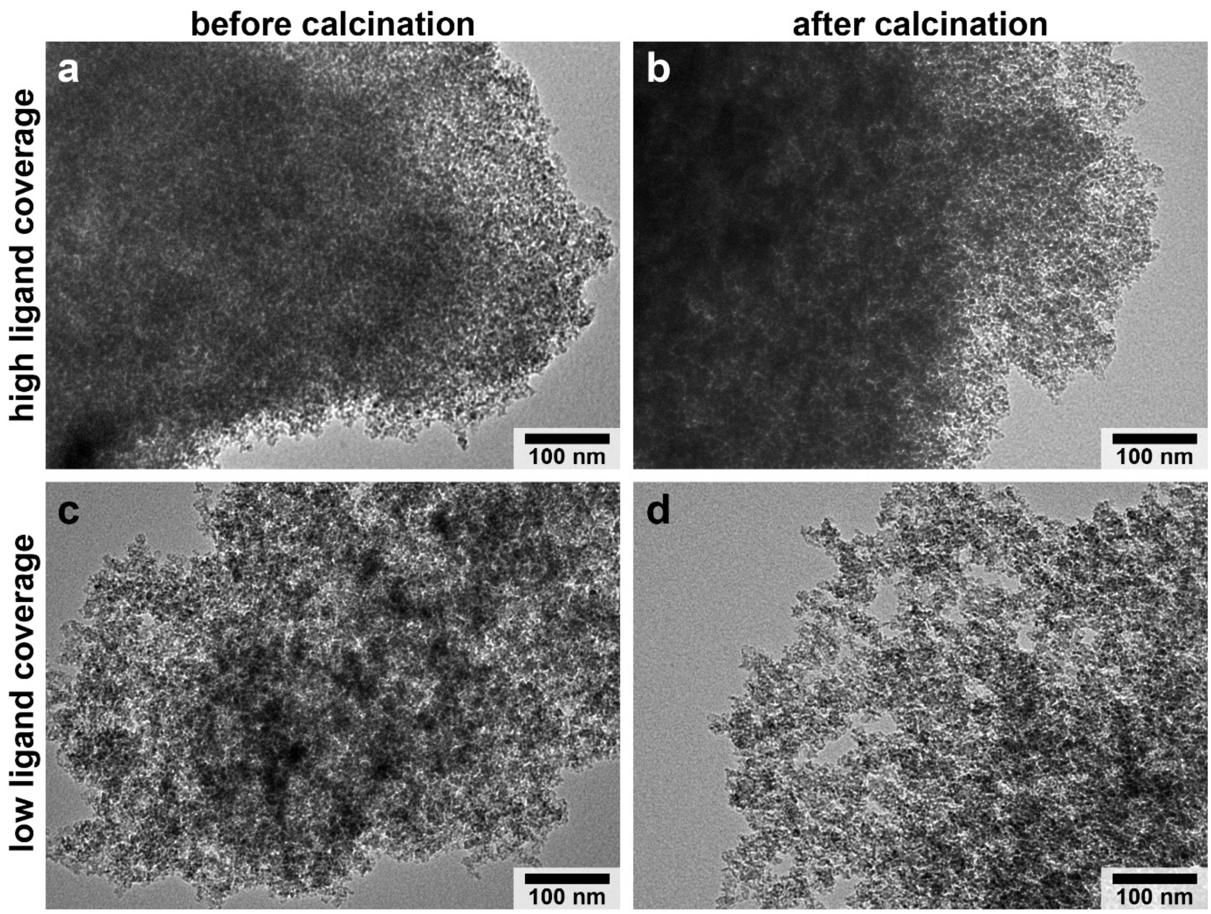

Supplementary Fig. 3. TEM images of parts printed from cages with high ligand coverage (**a,b**) and low ligand coverage (**c,d**), before (**a,c**) and after (**b,d**) calcination.

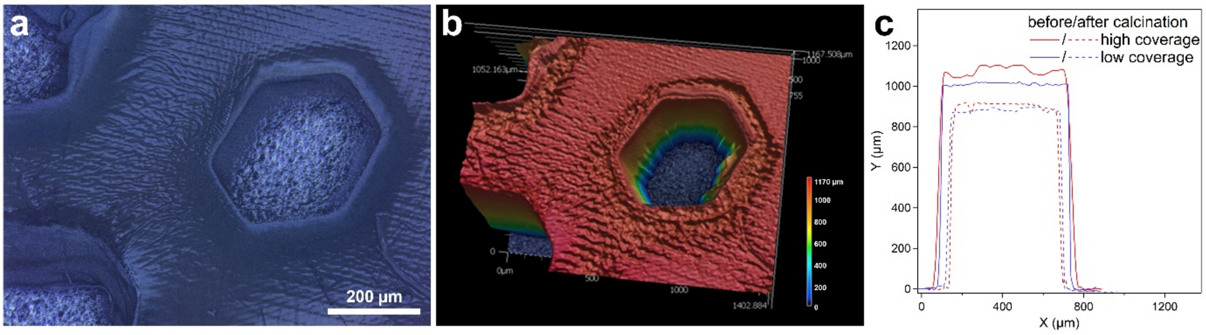

Supplementary Fig. 4. Confocal microscopy image (**a**) and 3D view (**b**) of a free-standing part with honeycomb structure (sample with high ligand coverage before calcination). (**c**) Line profiles across a strut of honeycomb structures printed from cages with high ligand coverage (red lines) and low ligand coverage (blue lines), before (continuous lines) and after (dashed lines) calcination.

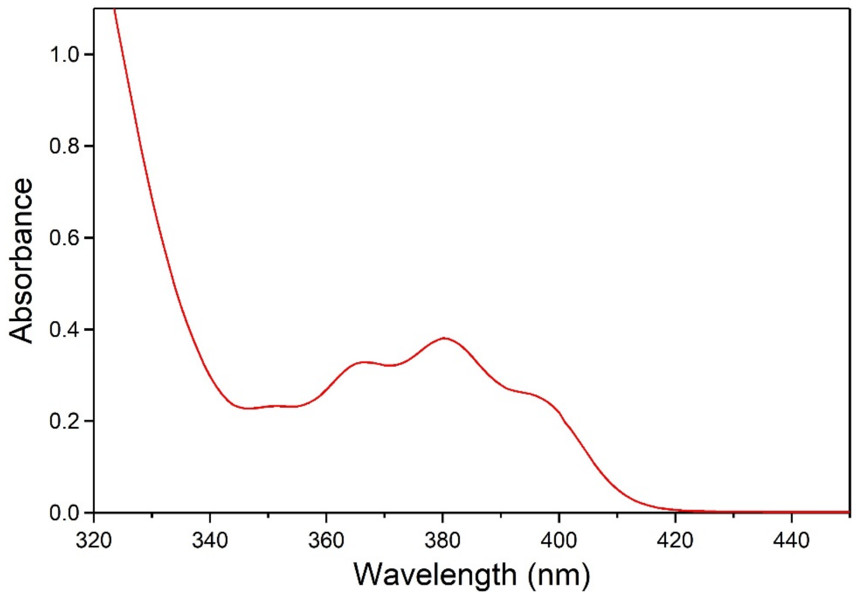

Supplementary Fig. 5. Absorption spectrum of TPO at 0.7 mM in ethanol:toluene (1:1 vol%).

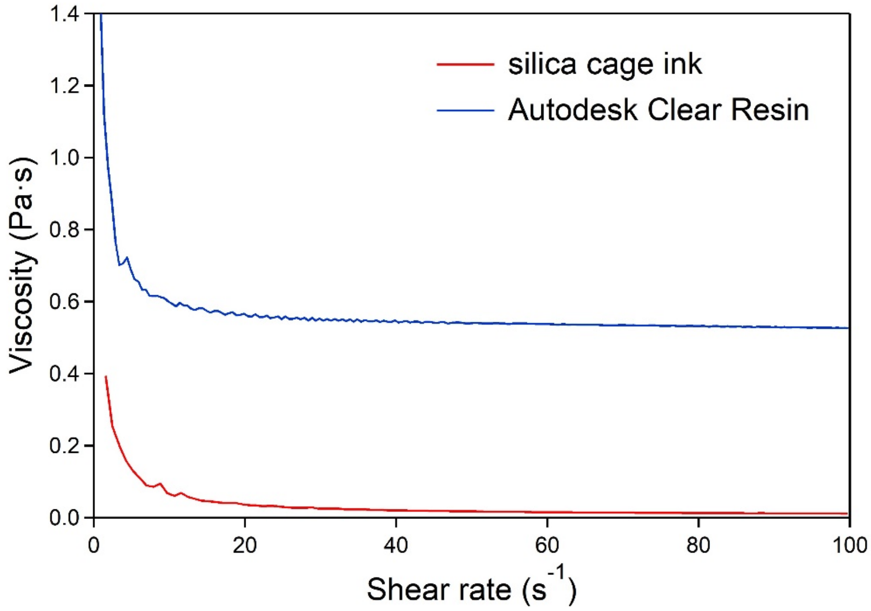

Supplementary Fig. 6. Viscosity of the silica cage ink (red line) and a commercial 3D printing resin (Autodesk Clear Resin, blue line) as a function of shear rate.

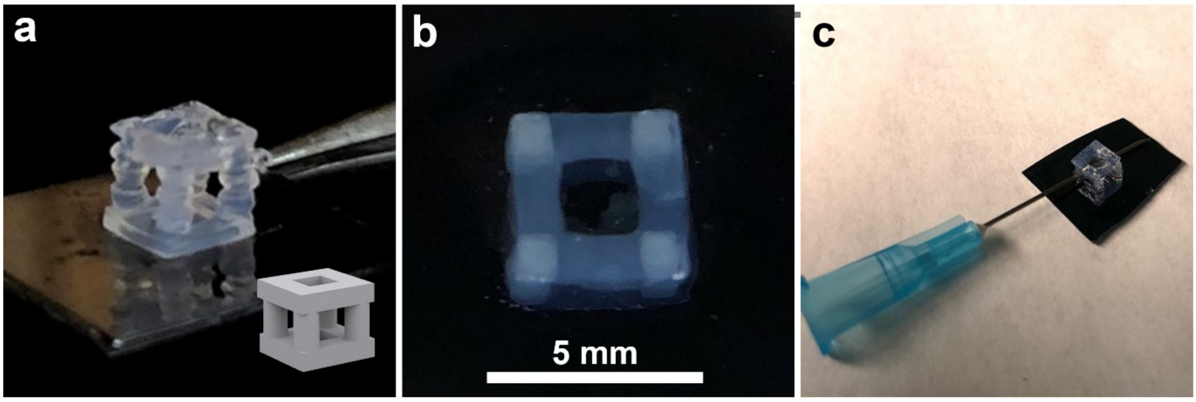

Supplementary Fig. 7. a-c, Photographs of a 3D printed structure with hanging features (inset in a: 3D model of the structure).

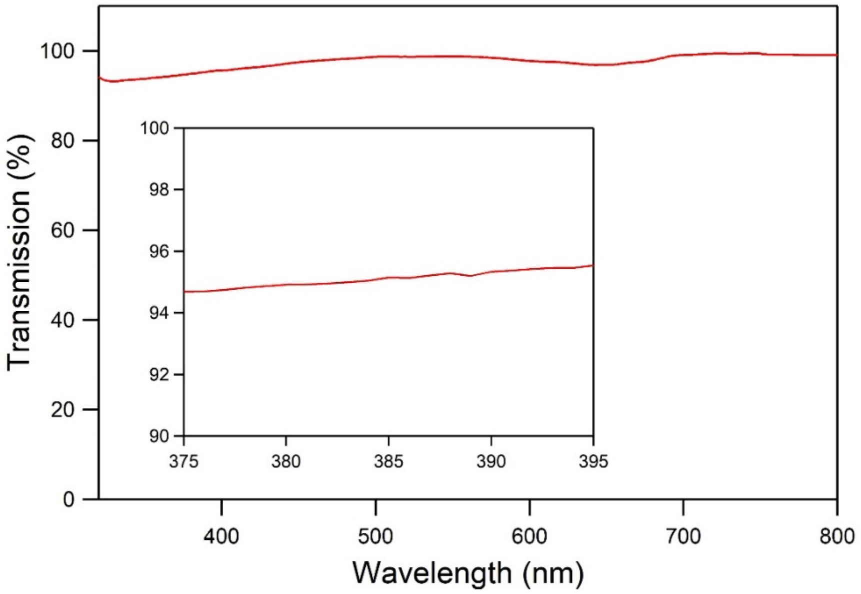

Supplementary Fig. 8. Transmission spectrum from a set-up with 1 cm pathlength through the as-prepared ink of the methacrylate functionalized silica cages without photoinitiator (inset: zoom-in to the DLP projector spectral range).

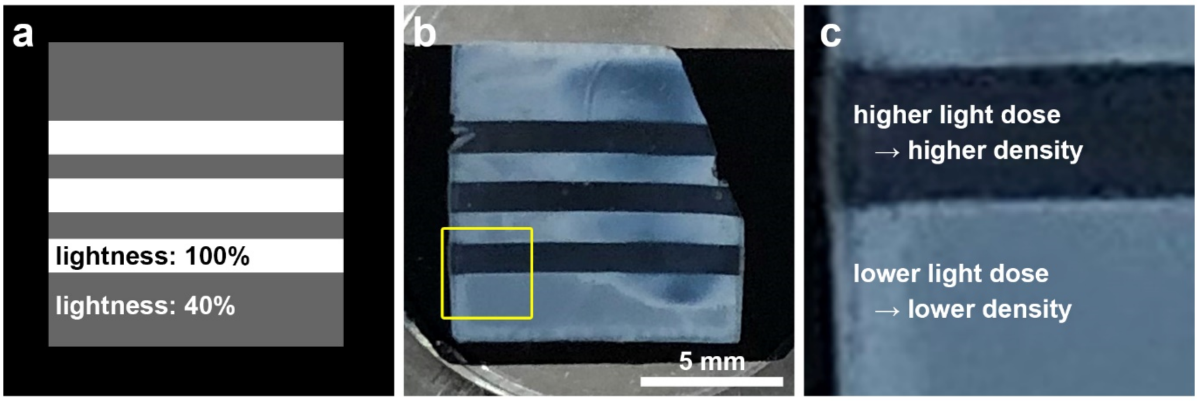

Supplementary Fig. 9. Greyscale pattern (**a**) and photograph of the resulting printed part (**b**). **c**, Zoom in to the region highlighted with a yellow square in b.

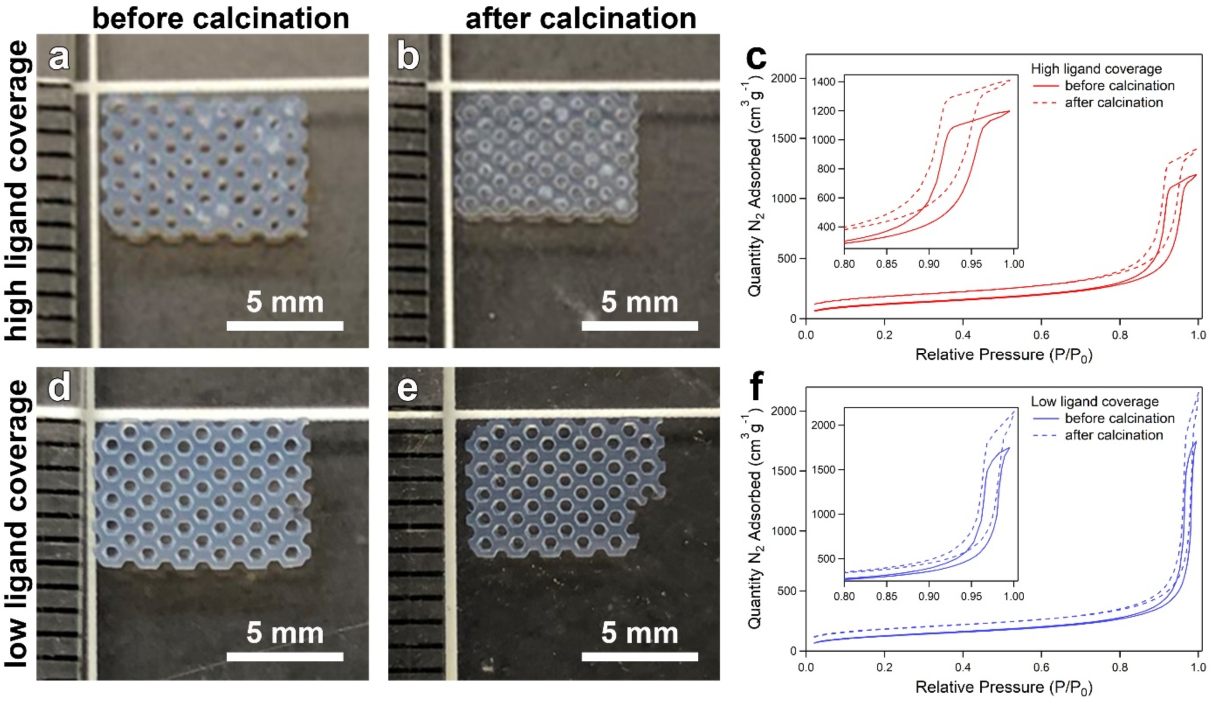

Supplementary Fig. 10. Photographs and nitrogen sorption measurements of parts printed from cages with high ligand coverage (**a-c**) and low ligand coverage (**d-f**). The comparison of the photographs before (a,d) and after (b,e) calcination demonstrates that the macroscopic shape of the parts is preserved after calcination (although some shrinkage takes place, see main text).

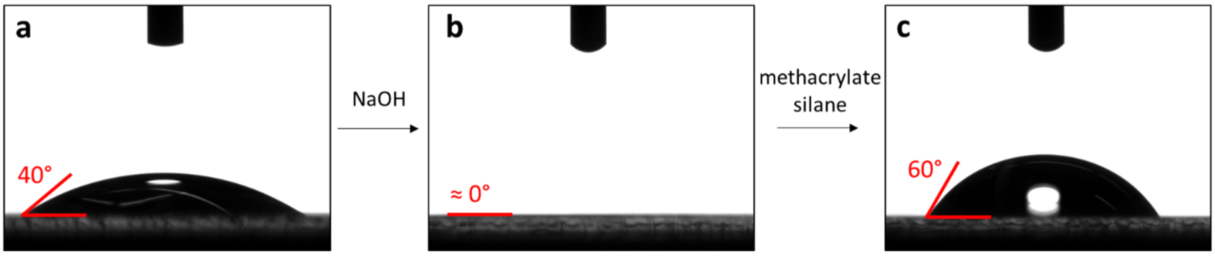

Supplementary Fig. 11. Contact angle measurements of a water droplet on a cover glass slide, as received (**a**), after 1 day in 0.2 M NaOH (**b**), and after functionalization with methacrylate silane (**c**). The NaOH treatment generates surface silanol groups resulting in a highly hydrophilic surface on top of which the water droplet fully spreads with a contact angle close to 0° (b). This activated surface promotes the bonding of methacrylate-silane, resulting in a hydrophobic surface as evidenced by an increase of the contact angle (c). The methacrylate groups on the surface can readily bond with the functionalized cages during the 3D printing process, enhancing the adhesion of printed parts on the substrate.

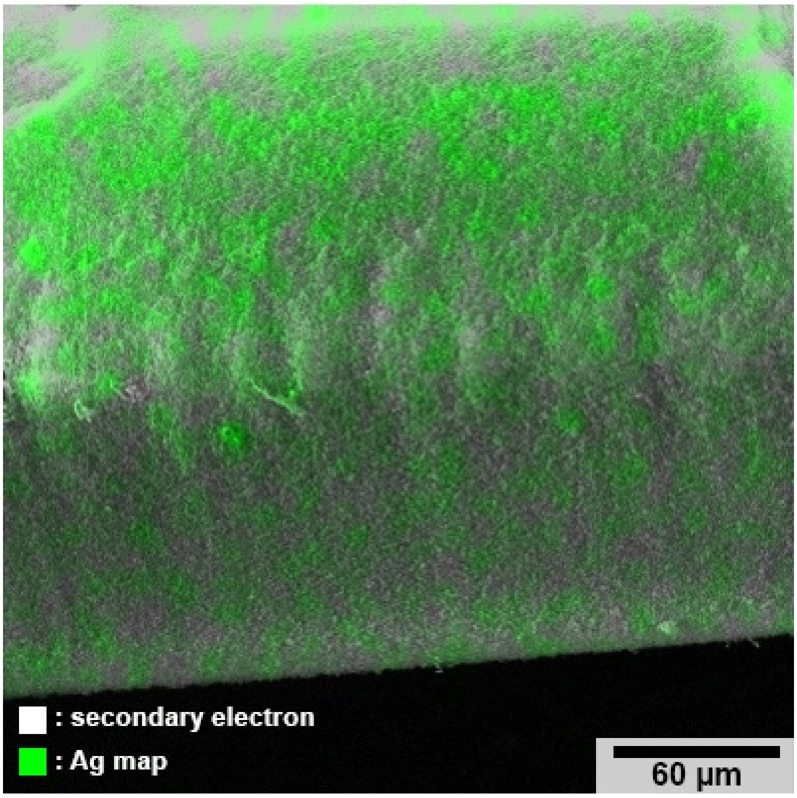

Supplementary Fig. 12. EDS analysis of the cross section from a silver slab printed within a silica part. The Ag map is overlaid with the SEM secondary electron image.

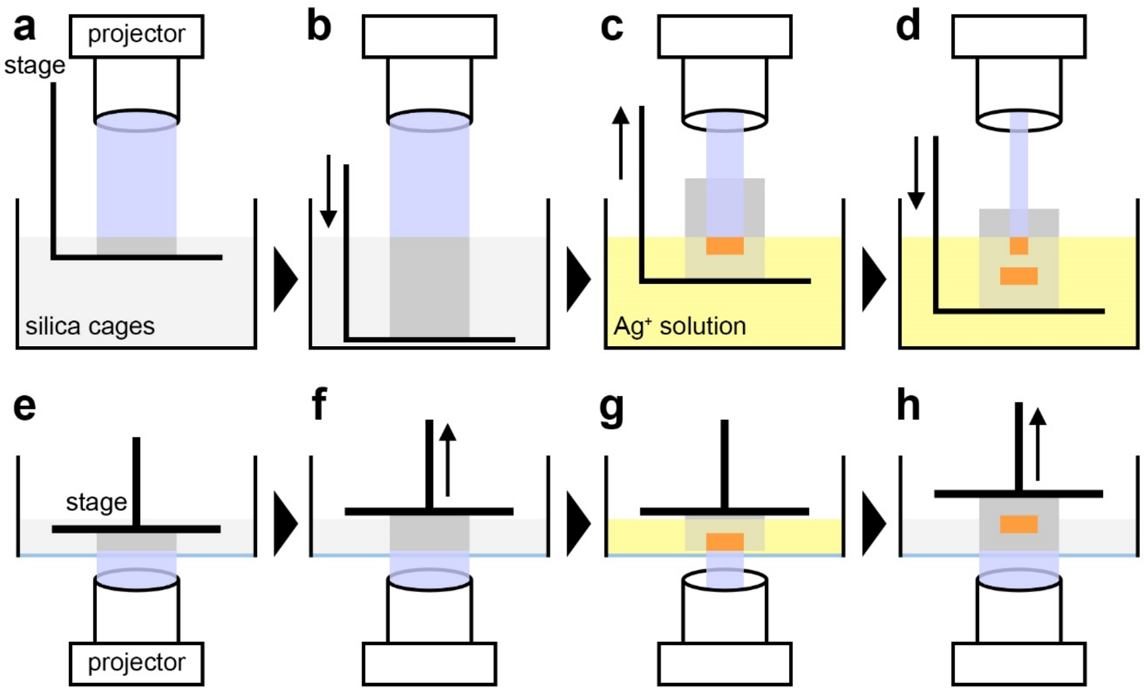

Supplementary Fig. 13. Illustration of different options for the implementation of internal 3D printing in either a top-down (**a-d**) or bottom-up (**e-h**) projection setup. In top-down, the host structure can be fully printed first (**a,b**), then progressively re-immersed in the vat containing the precursor solution of the guest material (**c,d**). In bottom-up, after printing a layer of the host structure (**e,f**), the vat ink is replaced for the printing of guest material within this layer (**g**), and changed again for the next host structure layer (**h**).

Supplementary Reference

1 Bressy, C., Ngo, V. G., Ziarelli, F. & Margaillan, A. New Insights into the Adsorption of 3-(Trimethoxysilyl)propylmethacrylate on Hydroxylated ZnO Nanopowders. *Langmuir* **28**, 3290-3297 (2012).
